# White matter fiber tracking directed by interpolating splines and a methodological framework for evaluation

**DOI:** 10.3389/fninf.2013.00013

**Published:** 2013-07-26

**Authors:** Are Losnegård, Arvid Lundervold, Erlend Hodneland

**Affiliations:** ^1^Neuroinformatics and Image Analysis Laboratory, Department of Biomedicine, University of BergenBergen, Norway; ^2^Kavli Research Centre for Aging and Dementia, Haraldsplass Deaconess HospitalBergen, Norway; ^3^Department of Radiology, Haukeland University HospitalBergen, Norway

**Keywords:** white matter, tractography, along-tract, orientation distribution function, fractional anisotropy, longitudinal data analysis, spline interpolation, aging neuroscience

## Abstract

Image-based tractography of white matter (WM) fiber bundles in the brain using diffusion weighted MRI (DW-MRI) has become a useful tool in basic and clinical neuroscience. However, proper tracking is challenging due to the anatomical complexity of fiber pathways, the coarse resolution of clinically applicable whole-brain *in vivo* imaging techniques, and the difficulties associated with verification. In this study we introduce a new tractography algorithm using splines (denoted Spline). Spline reconstructs smooth fiber trajectories iteratively, in contrast to most other tractography algorithms that create piecewise linear fiber tract segments, followed by spline fitting. Using DW-MRI recordings from eight healthy elderly people participating in a longitudinal study of cognitive aging, we compare our Spline algorithm to two state-of-the-art tracking methods from the TrackVis software suite. The comparison is done quantitatively using diffusion metrics (fractional anisotropy, FA), with both (1) tract averaging, (2) longitudinal linear mixed-effects model fitting, and (3) detailed along-tract analysis. Further validation is done on recordings from a diffusion hardware phantom, mimicking a coronal brain slice, with a known ground truth. Results from the longitudinal aging study showed high sensitivity of Spline tracking to individual aging patterns of mean FA when combined with linear mixed-effects modeling, moderately strong differences in the along-tract analysis of specific tracts, whereas the tract-averaged comparison using simple linear OLS regression revealed less differences between Spline and the two other tractography algorithms. In the brain phantom experiments with a ground truth, we demonstrated improved tracking ability of Spline compared to the two reference tractography algorithms being tested.

## Introduction

Diffusion-weighted MRI (DW-MRI) of the brain is a technique used for measuring white matter (WM) fiber architecture. Tractography (Mori et al., 1998; Basser et al., 2000) aims at reconstructing the fiber trajectories, and thus gives *in vivo* information about myelinated fiber bundle connections in the brain. Various diffusion parameters, e.g., fractional anisotropy (FA), mean diffusivity (MD), and radial diffusivity (RD), are used to quantify developmental or disease related white matter structural changes in the brain (Westlye et al., 2010; Lebel et al., 2012; Madden et al., 2012).

WM fiber tracking was originally based on the use of a voxel-wise diffusion tensor (DT) representation of the recorded data. The earlier form of DW-MRI was first introduced by Basser et al. (Basser and LeBihan, 1992; Basser et al., 1992). Later, Westin et al. (1999) showed various useful measures for tract analysis, e.g., the scalar FA measure. Although highly valuable, the use of a single tensor to represent the diffusion in a voxel has limitations. The coarse image resolution will give voxels that contain fibers of possibly different directions. The diffusion ellipsoid, reconstructed from the eigenvalues and eigenvectors of the tensor, is applicable to tractography in voxels with a single fiber direction, but contain deficient information in voxels with more than one fiber direction (Jones, 2011). A recent study (Jeurissen et al., 2012) estimated that 63–90% of WM voxels contain crossing fibers, meaning that the diffusion tensors provide insufficient information about fiber directions in the majority of WM voxels, and could therefore mislead a tractography method.

To solve the challenge of complex fiber configurations, more advanced (and time-consuming) acquisition schemes have been developed, such as diffusion spectrum imaging (DSI) (Wedeen et al., 2000; Tuch et al., 2001) and Q-ball imaging (Tuch, 2004). They are referred to as “high angular resolution diffusion imaging” (HARDI). In these techniques, the diffusion is measured at higher spatial density of the diffusion sensitizing gradients, and at higher *b*-values. These studies also introduced an alternative way to represent the diffusion signal, different from the classical diffusion tensor. The measured diffusion signal is now mapped to a function on a sphere, denoted the orientation distribution function (ODF), using a transformation proposed by Stejskal and Tanner (1965). The ODF either reflects the directional distribution of diffusion and is referred to as a diffusion ODF (dODF) (Tuch, 2004), or estimates the fiber directions, and is referred to as fiber ODF (fODF) (Descoteaux et al., 2009). Subsequent studies have shown that these transformation techniques improve tracking in DSI (Wedeen et al., 2008; Fernandez-Miranda et al., 2012), and recent investigations have shown that the use of ODFs may also improve fiber tracking in standard clinical acquisitions (Prckovska et al., 2010). However, most HARDI reconstruction techniques are resource demanding and time-consuming and seem not to be readily available for the clinics.

Several tracking methods have been tested on WM regions and myelinated fiber bundles (Mori et al., 1998; Basser et al., 2000; Mori and van Zijl, 2002; Parker et al., 2002; Wedeen et al., 2008; Reisert et al., 2011; Pontabry et al., 2013), where streamline methods (Basser et al., 2000; Wedeen et al., 2008) and “fiber assignment by continuous tracking” (FACT) (Mori et al., 1998) are the most commonly used. Alternative tracking methods have been developed, e.g., using the Fast Marching method (Parker et al., 2002; Campbell et al., 2005; Losnegård et al., 2011), global tracking in a Bayesian framework (Reisert et al., 2011) and using Markov chains (Pontabry et al., 2013), but state-of-the-art software in common use is typically based on the original ideas of linear line propagation techniques (Mori et al., 1998; Basser et al., 2000). Despite improved DW-MRI acquisitions with more expressive diffusion representations that allow complex fiber configurations, tractography still remains a challenging task, in particular for clinically feasible acquisition protocols (Besseling et al., 2012).

In this context, we are developing a new tractography algorithm inspired by the smooth nature of WM fiber bundles (Gössl et al., 2002; Jones, 2011; Catani and de Schotten, 2012). Standard tracking methods reconstruct piecewise linear trajectories that are subsequently smoothed. Instead of subsequent smoothing, our method smooths (by spline construction) the fibers iteratively, and we investigate the effect this approach has on the reconstructed trajectories. We first evaluate the method on DW-MRI data acquired at three different time points from eight healthy elderly people participating in a longitudinal study of cognitive aging, using a 1.5 T scanner and a clinically applicable acquisition protocol with 25 diffusion sensitizing directions. In the evaluation, we focus on the *corticospinal tract* (*CST*) and the trans-callosal *forceps minor* (*Fminor*) fiber bundles. The *CST* has been studied previously (Westlye et al., 2010; Lebel et al., 2012), reporting only a 0.02 reduction in tract-average FA for the age span from 40 to 80 years. In contrast, the FA decline in *Fminor* for the same age span was found to be 0.08 in Westlye et al. (2010) and 0.04 in Lebel et al. (2012), indicating less affected FA values during aging for the *CST* compared to the *Fminor* tract. Moreover, along-tract FA analysis in e.g., (Colby et al., 2012; Hodneland et al., 2012; Yeatman et al., 2012) revealed local FA variations that were not captured by simple grand mean FA across the tract. The two well-studied fiber bundles *CST* and *Fminor* were therefore chosen as a real-data tested for comparing our Spline method to other tracking methods. For further testing the performance of our algorithm we employed DW-MRI data from a brain phantom, designed for the MICCAI 2009 Fiber Cup with known ground truth (Poupon et al., 2008, 2010; Fillard et al., 2011; Côte et al., 2012), where a 3T MRI-scanner and 64 diffusion sensitizing directions had been used.

The rest of the paper is organized as follows. First we describe the orientation distribution function (dODF) in its probabilistic setting, and then we describe our Spline tracking algorithm using the voxel-wise dODF estimates. The streamline tracking algorithms which are compared to our Spline approach is then presented before more detailed anatomical descriptions of the selected fiber bundles *CST* and *Fminor* are provided. In the Materials and Methods section we also give a description of the MR imaging data and acquisition parameters being used for the experiments, and the linear mixed-effects model used in the assessment of longitudinal recordings. In the Results section we describe the outcome from processing the longitudinal healthy aging MRI-data, comparing Spline with the two other tracking methods, before processing results from the Fiber Cup test object recordings are given. In the last section we discuss our approach in a broader context and point to improvements and follow-up experiments for future work.

## Materials and methods

### Orientation distribution function

Orientation distribution functions (ODF) (Wedeen et al., 2000; Tuch et al., 2001; Descoteaux et al., 2007; Yeh et al., 2010) are computed from the original diffusion data and can be used to obtain voxelwise information about preferred diffusion directions. The dODF reconstruction technique used in this study is implemented in TrackVis (Wang et al., 2007; Wedeen et al., 2008). From the diffusion signal *S*, acquired at the diffusion sensitizing directions **q**, a probability density function (PDF) of spin displacements *p*(**r**) is computed:

(1) p(r)=ℱ{|S(q)|}(r),

where ℱ is the discrete Fourier transform and **r** is the 3D displacement vector. The dODF *w*(**v**) is then computed by projection of the data in the radial direction:

(2) $$w(v)=\int p(\rho v)\rho^{2}d\rho$$

where **v** is a unit 3D vector, ρ = |**r**| and ρ^2^ *d*ρ is the 3D volume element. The integral is computed as a discrete sum over the range ρ ∈ [0, 5] voxels in diffusion *r*-space. Then, *w*(**v**) is evaluated for a set of vectors at the vertices of a regular polyhedron. Local maxima of the dODFs are estimated subsequently and used as voxelwise fiber orientations.

**Figure 1:**
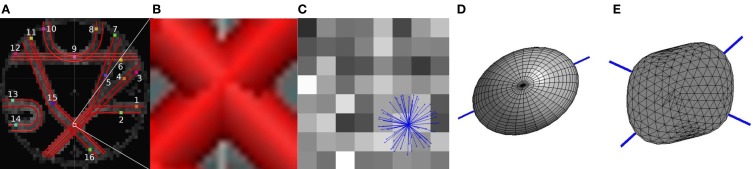
**(A)** The diffusion phantom with fibers indicated with red tubes on top of the FA map. ROIs used to identify the fibers are indicated with a colored square and a number. **(B)** The ground truth fiber orientations in one voxel. **(C)** Sixty-four squares displaying the diffusion values from the diffusion sensitizing directions, indicated with blue vectors. **(D)** The diffusion ellipsoid computed from the diffusion tensor. **(E)** The dODF. Blue lines in **(D)** and **(E)** indicate the fiber orientations obtained from the diffusion tensor and the dODF, respectively.

Figure 1 displays the fiber configuration for a single voxel (Figures 1A,B), the diffusion signal (Figure 1C), the corresponding diffusion ellipsoid (Figure 1D), and the dODF (Figure 1E). Clearly, the dODF representation contains significantly more detailed information about the local water diffusion than the diffusion tensor ellipsoid, and is able to approximate two fiber directions instead of only one (see also Figure 7).

### Spline tracking

Our spline tracking algorithm is inspired by the smooth nature of WM fibers (Gössl et al., 2002; Jones, 2011; Catani and de Schotten, 2012). To achieve a similar smoothness iteratively, we make the use of cubic b-splines (Gerald and Wheatley, 1999). This has two main advantages. Firstly, the propagation is directed by the dODFs along the splines, instead of along the piecewise linear segments. Secondly, noisy voxels may terminate fibers too early using streamline tracking. The use of splines iteratively during the tracking can reduce the impact of such noisy voxels. The cubic b-splines implemented here require at least four control points, and returns a smooth curve between the middle two points. Given four control points $p_{0}^{\left(i\right)}=\left(x_{0}^{\left(i\right)},y_{0}^{\left(i\right)},z_{0}^{\left(i\right)}\right),\ldots ,p_{3}^{\left(i\right)}=\left(x_{3}^{\left(i\right)},y_{3}^{\left(i\right)},z_{3}^{\left(i\right)}\right)$, the first element of the position vector $S^{\left(i\right)}(u)=\left(s_{x}^{\left(i\right)}(u),s_{y}^{\left(i\right)}(u),s_{z}^{\left(i\right)}(u)\right)$ becomes

(3) $$s_{x}^{\left(i\right)}(u)=\frac{1}{6}\left[u^{3},u^{2},u,1\right]\;[\begin{array}{cccc} -1 & 3 & -3 & 1\\ 3 & -6 & 3 & 0\\ -3 & 0 & 3 & 0\\ 1 & 4 & 1 & 0 \end{array}]\;[\begin{array}{c} x_{0}^{\left(i\right)}\\ x_{1}^{\left(i\right)}\\ x_{2}^{\left(i\right)}\\ x_{3}^{\left(i\right)} \end{array}]$$

where *i* indicates the iteration number and *u* ∈ {0 ν, 1ν, 2ν, …, 1}. We choose ν = 0.01. The two other components of *s*^(*i*)^_*y*_(*u*) and *s*^(*i*)^_*z*_(*u*) are computed similarly by exchanging *x*^(*i*)^_0, …, 3_ with *y*^(*i*)^_0, …, 3_ and *z*^(*i*)^_0, …, 3_, respectively. A fiber can then be represented by a set of coordinates *S* = {**S**^(*i*)^}^*n*^_*i* = 1_.

**Figure 2:**
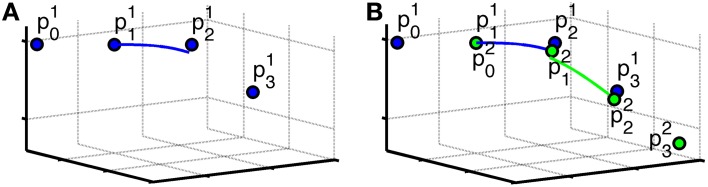
**Illustration of the definition of points in **Spline**. (A)** The initial points of a fiber (the control points), **p**^(1)^_0_, …, **p**^(1)^_3_, and the resulting spline (blue curve). **p**^(1)^_1_ is the seed point, **p**^(1)^_2_ is found by going a step-length in the direction of a local maxima in **p**^(1)^_1_, **p**^(1)^_0_ is found by going in the opposite direction, and **p**^(1)^_3_ is found by going a step-length in the direction of the local maxima most closely to the incoming direction in **p**^(1)^_2_. **(B)** The second iteration. **p**^(2)^_0_, …, **p**^(2)^_3_, and the resulting spline. The points marked with **p**^(1)^_0_, …, **p**^(1)^_3_ in blue are the points from the first iteration. Note that **p**^(2)^_1_ from this iteration and **p**^(1)^_2_ from the previous iteration are in this case not the same point. The same is the case for **p**^(2)^_2_ from this iteration and **p**^(1)^_3_ from the previous iteration. The resulting splines are shown for iteration 1 (blue curve) and iteration 2 (green curve).

Algorithm Spline, given below as pseudo-code, demonstrates the procedure. As in Equation (2), let *w*(**x**, **û**) be the dODF in the voxel coordinate **x** in direction **û**. Based on the seed point, **p**^(1)^_1_ we locate **p**^(1)^_0_, **p**^(1)^_2_, and **p**^(1)^_3_. The point **p**^(1)^_2_ is found by a forward step *h* in the direction of a local maximum of the dODF, *w*(**p**^(1)^_1_, ${\hat{Q}}_{1}$) (cf. Figure 2A). **p**^(1)^_0_ is found by a backward step of length *h* in the opposite direction. **p**^(1)^_3_ is found by propagating a step-length from **p**^(1)^_2_ in direction of ${\hat{Q}}_{2}$, which is the direction of the local maximum of the dODF in **p**^(1)^_2_ closest aligned with the incoming direction, in the sense of the largest dot product. In general, we find

(4) $${\hat{Q}}_{k}=\text{arg}\underset{{\hat{q}}_{k}\in M}{\max}{\hat{Q}}_{k\;-\;1}\cdot {\hat{q}}_{k},$$

where $M=\{{\hat{q}}_{k}\}=\text{arg local}\max_{\hat{u}}w(p_{k},\hat{u})$ is the set of local maxima directions of *w* at **p**^(*i*)^_*k*_. Using Equation (3) we obtain a spline that is valid between **p**^(1)^_1_ and **p**^(1)^_2_. In the next iteration, we define **p**^(2)^_0_ to be **p**^(1)^_1_ and **p**^(2)^_1_ as the end point of the spline computed in the first iteration, which may not be equal to **p**^(1)^_2_. The next point **p**^(2)^_2_ is found a step-length from **p**^(2)^_1_ in the direction of the local maximum at **p**^(2)^_1_ closest to the incoming direction. In a similar way, **p**^(2)^_3_ is found by considering the dODF in **p**^(2)^_2_, and going a step in the local maximum closest to the incoming direction. Both local maxima are found using Equation (4). This procedure is repeated iteratively for new points.

The maximum number of iterations *n* is not pre-defined. However, when the fiber reaches a voxel with FA ≤ 0.15 or the fiber takes a sharp turn, the iterations stop. A sharp turn is defined by a large dot product between the fiber direction of consecutive iterations, when

(5) $$\theta =\arccos\left(\frac{\left(p_{2}^{\left(i\right)}-p_{1}^{\left(i\right)}\right)\cdot \left(p_{2}^{\left(i+1\right)}-p_{1}^{\left(i+1\right)}\right)}{\left|\left(p_{2}^{\left(i\right)}-p_{1}^{\left(i\right)}\right)\right|\left|\left(p_{2}^{\left(i+1\right)}-p_{1}^{\left(i+1\right)}\right)\right|}\right)\geq \theta_{\max}.$$

We used θ_max_ = 60°.

**Algorithm Spline:** 

Let **p**^(1)^_1_ be a seed point, and ${\hat{Q}}_{k}$ and ${\hat{Q}}_{k+1}$ satisfy (4). *h* is the step-length.
$p_{0}^{\left(1\right)}=p_{1}^{\left(1\right)}-{\hat{Q}}_{1}h$
**p**^(1)^_1_ is a seed point
$p_{2}^{\left(1\right)}=p_{1}^{\left(i\right)}+{\hat{Q}}_{1}h$
$p_{3}^{\left(1\right)}=p_{2}^{\left(i\right)}+{\hat{Q}}_{2}h$
For *i* = 2: *n*
**p**^(*i*)^_0_ = **p**^(*i* − 1)^_1_
**p**^(*i*)^_1_ = end point of **S**^(*i* − 1)^
$p_{2}^{\left(i\right)}=p_{1}^{\left(i\right)}+{\hat{Q}}_{1}h$
$p_{3}^{\left(i\right)}=p_{2}^{\left(i\right)}+{\hat{Q}}_{2}h$
Compute *S*^(*i*)^, as described in (3).
If θ ≥ 60° or FA ≤ 0.15, then Break
End

Since we update the points as we move along the fiber, the splines are not continuous, as we see in Figure 2. When the full trajectory is extracted, we select points along the tract and compute a smooth tract using cubic b-splines similarly to Equation (3), now only using every 20 fiber coordinate.

### Comparative streamline tracking

We performed the comparative streamline tracking using two available state-of-the-art reconstruction methods in TrackVis (Wang et al., 2007; Wedeen et al., 2008), using dODFs and DTs (denoted DTKODF and DTKTensor, respectively). Standard streamline tractography is based on the Frenet equation:

(6) $$\frac{dr(s)}{ds}=t(s).$$

Here, **r**(*s*) is the curve, *s* the arc length and **t**(*s*) the unit tangent vector of the curve at *s*. The equation is solved iteratively using Euler integration. In DTKODF, **t**(*s*) equals the local maximum of the dODF most closely aligned with the incoming direction (as in Equation 4), whereas in DTKTensor, **t**(*s*) is the principal eigenvector of the DT.

### Imaging data

In the cognitive aging project at the University of Bergen (Westlye et al., 2011; Ystad et al., 2011; Hodneland et al., 2012) longitudinal and multimodal MRI data (3D T1-weighted, resting state BOLD fMRI, and DW-MRI) from more than 100 healthy elderly volunteers has been acquired at three different study waves: 2005 (wave1), 2008/9 (wave2), and 2011/12 (wave3) on a 1.5 T GE Signa Excite scanner with a standard eight channel receive only head coil. Informed consent was obtained from all subjects. We have randomly chosen eight subjects (6 females; mean age at inclusion: 58 years, range 52–66) from this cohort for our analysis. The DW-MRI data were acquired with a 2D EPI SE sequence, 0.94 × 0.94 × 4 mm^3^ voxels and 25 diffusion sensitizing directions (*b* = 1000 s/mm^2^) in addition to five *b*_0_ volumes, with 26 axial slices TR/TE/FA/NAC = 7900 ms/97.1 ms/90°/2 in wave1; 25 axial slices TR/TE/FA/NAC = 7900 ms/104.8 ms/90°/2 in wave2, and 25 axial slices TR/TE/FA/NAC = 7900 ms/110.5 ms/90°/2 in wave3. Scanning time for the DW-MRI was about 7 min The data was resampled to 2 × 2 × 2 mm^3^ isotropic voxels using FSL's resampling algorithm applyxfm4D (Jenkinson and Smith, 2001).

To further validate our method, we used the MICCAI 2009 Fiber Cup phantom dataset (Poupon et al., 2008, 2010; Fillard et al., 2011). It resembles three coronal brain slices and was made of hydrophobic acrylic fibers with proportions similar to myelinated fibers. DW-MRI of the phantom was acquired with 3 × 3 × 3 mm^3^ isotropic voxels and 64 diffusion sensitizing directions (*b* = 1500 s/mm^2^) in addition to one *b*_0_ volume on a 3 T Siemens Tim Trio MRI system, equipped with a whole body gradient coil (40 mT/m, 200 T/m/s) and a 12-channel receive only head coil, used in combination with the whole body transmit coil of the MRI system. The acquisitions were performed using a single-shot diffusion-weighted twice refocused spin echo echoplanar pulse sequence, with volume size 64 × 64 × 3 voxels TR/TE/FA/NAC = 5000 ms/94 ms/65°/2.

### Selection of fiber tracts

**Figure 3:**
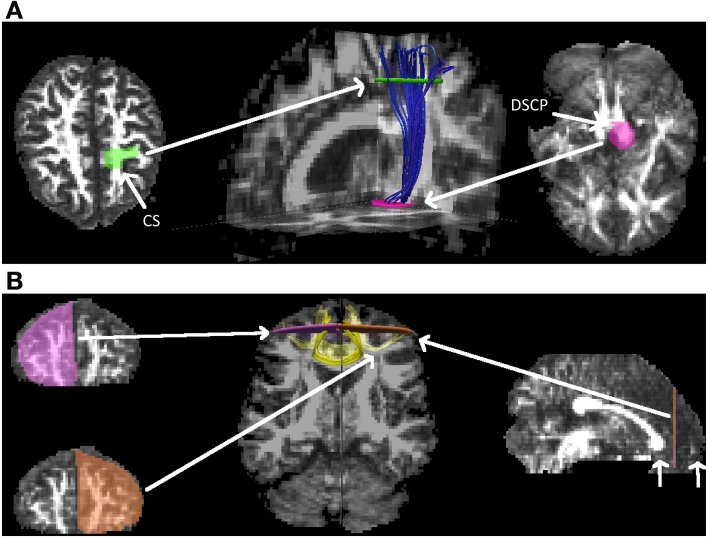
**The two fiber tracts, *CST* and *Fminor*, subject to quantitative analysis. (A)** The *corticospinal tract* (*CST*) in one of the subjects, extracted by using one ROI (pink surface) on the cerebral peduncle at the level of the decussation of the superior cerebellar peduncle (DSCP) and one ROI (green surface) at the central sulcus (CS) after the bifurcation to the motor and sensory cortex. **(B)** The *forceps minor* (*Fminor*) in one of the subjects, extracted in a coronal slice by using two ROIs (pink and brown surfaces) at the mid-point between the anterior tip of the frontal lobe and the anterior edge of the genu of the corpus callosum (indicated with short white arrows), in each hemisphere.

For the experiments on real data, we have chosen to study the *corticospinal tract* (*CST*), carrying information from the cerebral cortex to the spinal cord, and the *forceps minor* (*Fminor*), also named *forceps anterior*, which are trans-callosal fibers curving forward from the *genu* of the *corpus callosum* into the frontal lobes, carrying inter-hemispheric information. Both tracts were extracted for each subject at each of the three study waves (see above) by defining ROIs manually according to Wakana et al. (2007). For the *CST*, one ROI was drawn on the cerebral peduncle at the level of the decussation of the superior cerebellar peduncle. After displaying the fibers intersecting this ROI, the central sulcus and the projection to the motor cortex were identified and a ROI was delineated in the axial slice right after the bifurcation to the motor cortex and sensory cortex. The *CST* was defined in our experiments as the fibers intersecting these two ROIs (cf. Figure 3A). As mentioned in Wakana et al. (2007), the challenge with *CST* fibers continuing into the opposite brain hemisphere is solved by cutting the fibers at the inferior ROI. *Fminor* was extracted by drawing two ROIs in a coronal slice at the mid-point between the anterior tip of the frontal lobe and the anterior edge of the genu of the corpus callosum, one in each hemisphere, delineating the entire frontal lobe (cf. Figure 3B).

### Using linear mixed-effects models to assess differences between tracking methods on longitudinal data

For each of the tracts *CST* and *Fminor*, and each tracking method DTKODF, DTKTensor, and Spline, a linear mixed-effects (LME) model was fitted to the data, i.e.,

(7) $${\text{FA}}_{ij}=\beta_{0}+\beta_{1}{\text{Age}}_{ij}+(b_{0i}+b_{1i}{\text{Age}}_{ij})+\epsilon_{ij}.$$

Here, *FA*_*ij*_, the continuous *response variable* in the model, is mean FA in given tract and for given tracking method in subject *i* (*i* = 1, …, *N*) at wave *j* (*j* = 1, …, *n*_*i*_). In our case, we have *N* = 8 and three waves with complete data, i.e., *n*_*i*_ = 3 for all *i*. Age_*ij*_ is age (in years) of subject *i* at wave *j*, and a *predictor variable* in the model. The model parameters β_0_ and β_1_ are the *fixed effects* parameters. The variables *b*_0*i*_ and *b*_1*i*_ (*i* = 1, …, *N*) are the *random effects* parameters, assumed to be normally distributed with zero mean. They denote individual deviations in intercept and slope, respectively, from the group-level fixed effect. Finally, the random residual errors ∈_*ij*_ are assumed to be independent and normally distributed with zero mean and constant variance σ^2^_∈_. In the LME parameter estimation we used the restricted maximum likelihood (REML) criterion. Model Equation (7) allows for correlation among measurements obtained from the same subject at different waves after the fixed (population) effects have been taken into account.

**Figure 4:**
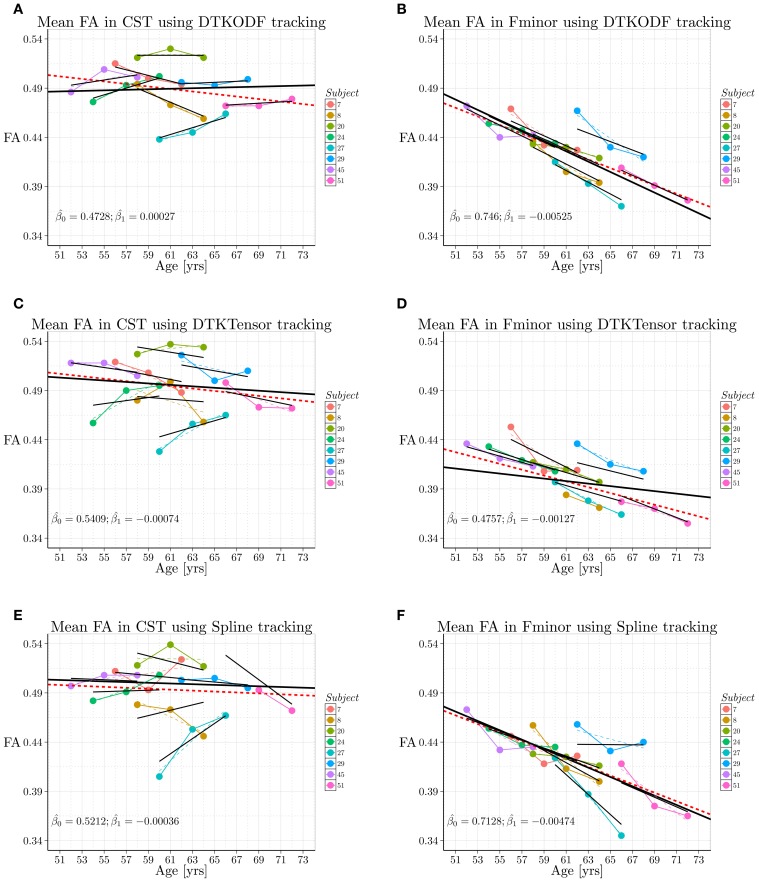
**Spaghetti plots from longitudinal analysis (wave1 ~2005, wave2 ~2008/9, wave3 ~2011/12) of tract grand mean FA (***y***-axis) for each of the eight subjects selected from the cohort**. Age of subject (*x*-axis) is given in years. Left column: *Corticospinal tract*. Right column: *Forceps minor*. Upper row: DTKODF tracking. Middle row: DTKTensor tracking. Lower row: Spline tracking. In each panel **(A–F)** the fixed effects estimates ($\hat{\beta_{0}}$ and $\hat{\beta_{1}}$) are given. The colored, thin broken lines are the subject-specific linear regression line for the three measurements. The short line segments in black represents the linear mixed-model fit (random effects) for each of the eight subjects, using lmer(). The black fat line, spanning across the full age range in the sample, is the population fixed effects estimate. The broken red line represents the (naive) simple linear regression OLS model, using lm(), where all measurements are regarded independent. Numerical summary results are given in Tables 2, 3.

We also compared the LME-model fits with simple linear OLS regression, assuming (naively) independence of between and within-subject measurements over time. The LME-model incorporates two sources of variation: a biological variation due to subjects, and a within-subject variability. In the comparison of tracking methods, we were interested in their influence on the fixed effect parameter estimates, on the variation between subjects, and on variation within subjects (residuals). For the longitudinal analysis we used R 3.0.0 (www.r-project.org) with the lmer() function of the lme4 package (http://lme4.r-forge.r-project.org) for LME model fitting, the ggplot2 package for making graphics (cf. Figure 4), and the stargazer package for generating tables (cf. Tables 2, 3).

## Results

### Tract-averaged and along-tract analysis in human DW-MRI

In our evaluation study we have examined longitudinal changes between wave1, wave2, and wave3 in the well-defined *CST* and *Fminor* fiber bundles. For the along-tract FA analyses, we employed the Colby MATLAB toolbox (Colby et al., 2012). Tracking was performed with step-length *h* = 1 mm (cf. Algorithm Spline), maximum angle θ = 60°, and minimum FA threshold at 0.15. The results for *CST* and *Fminor* are presented in Table 1, listing mean and standard deviations across all subjects. Table 1 shows that FA values of *CST* extracted by the different tracking methods in general has stable values over time. DTKTensor resulted in the overall highest mean FA values across the waves, followed by DTKODF and Spline. Averaging the standard deviations, DTKODF had the lowest standard deviations, followed by Spline and DTKTensor (both 0.029). The *Fminor* tract showed slightly decreasing FA values during time. For this tract, DTKODF resulted in the overall highest mean FA values, followed by Spline and DTKTensor. Also in this case, DTKODF had the lowest standard deviation on average, followed by Spline and DTKTensor. To quantify the changes in FA values across the three study waves, the absolute yearly changes are listed in the two last columns. Spline and DTKODF *CST* FA values were slightly increasing, while DTKTensor tracking gave slightly decreasing FA values. The *Fminor* FA values were decreasing the most for Spline, followed by DTKODF and DTKTensor tracking, where the Spline tracking method resulted in a mean reduction of 0.006 FA units per year, or about 1.4% yearly reduction with respect to a mean age of 58 years at baseline.

**Table 1:** ***CST* and *Fminor* FA values (mean and standard deviation) obtained with each of the tracking methods across the eight subjects for each of the three study waves**.

**Method**	**Wave1—2005**		**Wave2—2008/9**		**Wave3—2011/12**		**Yearly change (mean)**	
	***CST***	***Fminor***	***CST***	***Fminor***	***CST***	***Fminor***	***CST***	***Fminor***
DTKODF	0.487 (0.026)	0.444 (0.024)	0.489 (0.026)	0.421 (0.022)	0.490 (0.021)	0.410 (0.027)	0.001	−0.006
DTKTensor	0.494 (0.036)	0.397 (0.064)	0.497 (0.025)	0.401 (0.020)	0.491 (0.026)	0.391 (0.023)	−0.001	−0.001
Spline	0.482 (0.036)	0.444 (0.019)	0.494 (0.025)	0.414 (0.022)	0.491 (0.026)	0.407 (0.035)	0.002	−0.006

Mean yearly change (last column) was defined as the average yearly change between two successive waves, i.e., $\frac{1}{2}\left[\frac{{\bar{FA}}_{wave2}-{\bar{FA}}_{wave1}}{3}+\frac{{\bar{FA}}_{wave3}-{\bar{FA}}_{wave2}}{3}\right]$.

The wave-by-wave summary statistics (cf. Table 1) was supplied by more detailed longitudinal data analysis in terms of linear mixed-effects model fitting as explained in the Materials and Methods section. Initially, an exploratory visual analysis was performed by producing spaghetti plots of the tract-averaged FA values versus subject age at each examination time point, separately for each tract and for each tracking method. In Figure 4 the tract-averaged FA values for wave1, wave2, and wave3, are connected with color-coded line segments for a given subject to indicate subject-specific change over time.

From Figure 4 an immediate observation is the age-resistant and slightly higher FA values in *CST* compared to *Fminor*. For the *CST*, the three tracking methods performed qualitatively very similar with a rather stable FA value at about 0.49, and where subject 27 was an outlier with very low FA values in all tracking methods and subject 20 had consistently high FA values. Also for the *Fminor*, the methods performed qualitatively similar showing (on average) a clear reduction in FA over time, and where subject 29 seemed to be a consistent outlier with generally higher FA values for age in all tracking methods. However, more differentiating results could be obtained using the LME-modeling approach, enabling detailed quantitative assessment both at the group level and at the subject level. A summary of this analysis, together with simple OLS regression, is given in Tables 2, 3. A striking finding is the discrepancy between the linear trend in FA values obtained with simple OLS linear regression and the fixed-effect (population) regression line obtained with LME modeling. For the *CST* using DTKODF tracking, we actually found a slight linear increase in the fixed-effect fitting of FA during aging with a slope +0.00027 (SE 0.0012), while the OLS linear regression gave a negative slope of −0.0013 (SE 0.0010). Moreover, mean FA in *CST* using Spline tracking assessed with LME model-fitting gave comparable slope as with simple OLS fitting, −0.00036 and −0.00047, respectively, while the intercepts were 0.52122 and 0.52213, respectively. Thus, the LME-model predicts slightly higher mean FA in the age-range 51–73 years compared to simple OLS regression. Finally, the Spline tracking method together with LME model-fitting yielded the highest mean FA of the *CST* in elderly subjects (>65 years) compared to the other tracking methods. Regarding the *Fminor* tract, both DTKODF tracking and Spline tracking gave the largest decrease in mean FA with age (fixed-effects slopes −0.00525 and −0.00474, respectively), while DTKTensor tracking yielded a more moderate change in mean FA over time with a fixed-effects slope of −0.00127.

**Table 2:** ***CST*—Ordinary least squares lm() and linear mixed-model regression lmer() results**.

	**Dependent variable**					
	**Mean FA in *CST***					
	**OLS**			**Linear mixed-effects**		
	**(DTKODF)**	**(DTKTensor)**	**(Spline)**	**(DTKODF)**	**(DTKTensor)**	**(Spline)**
Constant—$\hat{\beta_{0}}$	0.567^***^	0.571^***^	0.522^***^	0.473^***^	0.541^***^	0.521^***^
	(0.060)	(0.073)	(0.082)	(0.076)	(0.088)	(0.132)
Age—$\hat{\beta_{1}}$	−0.001	−0.001	−0.0005	0.0003	−0.001	−0.0004
	(0.001)	(0.001)	(0.001)	(0.001)	(0.001)	(0.002)
Observations	24	24	24	24	24	24
R^2^	0.072	0.047	0.006			
Adjusted R^2^	0.030	0.004	−0.040			
Log likelihood				56.333	49.295	45.344
Akaike inf. crit.				−100.666	−86.589	−78.689
Bayesian inf. crit.				−93.597	−79.521	−71.621
Residual std. error (*df* = 22)	0.023	0.028	0.032			
F statistic (*df* = 1; 22)	1.719	1.093	0.126			

*** p < 0.01.

**Table 3:** ***Fminor*—Ordinary least squares lm() and linear mixed-model regression lmer() results**.

	**Dependent variable**					
	**Mean FA in *Fminor***					
	**OLS**			**Linear mixed-effects**		
	**(DTKODF)**	**(DTKTensor)**	**(Spline)**	**(DTKODF)**	**(DTKTensor)**	**(Spline)**
Constant—$\hat{\beta_{0}}$	0.693^***^	0.579^***^	0.690^***^	0.746^***^	0.476^**^	0.713^***^
	(0.045)	(0.100)	(0.056)	(0.039)	(0.190)	(0.077)
Age—$\hat{\beta_{1}}$	−0.004^***^	−0.003^*^	−0.004^***^	−0.005^***^	−0.001	−0.005^***^
	(0.001)	(0.002)	(0.001)	(0.001)	(0.003)	(0.001)
						
Observations	24	24	24	24	24	24
R^2^	0.614	0.132	0.508			
Adjusted R^2^	0.596	0.092	0.485			
Log likelihood				60.760	40.908	55.635
Akaike inf. crit.				−109.520	−69.817	−99.269
Bayesian inf. crit.				−102.452	−62.749	−92.201
Residual std. error (*df* = 22)	0.018	0.039	0.022			
F statistic (*df* = 1; 22)	34.979^***^	3.333^*^	22.688^***^			

* p < 0.1;

** p < 0.05;

*** p < 0.01.

The individual linear random-effects estimates had a larger variation in slope and intercept for the Spline tracking method (Figures 4E,F) compared to the two other methods. In the order of DTKODF, DTKTensor, and Spline tracking, the random effects summary statistics are given in Table 4.

**Table 4:** ***CST* and *Fminor*—Tracking-dependent random effects estimates in (7) using lmer()**.

**Tract**	**Random effect parameter**	**Tracking method**		
		**DTKODF**	**DTKTensor**	**Spline**
**CST**				
	$\text{Var}(\hat{b_{0}})$	3.8274 ×10^−2^	3.3341 × 10^−2^	1.1095 × 10^−1^
	$\text{Var}(\hat{b_{1}})$	9.8661× 10^−6^	7.1295 × 10^−6^	2.2747× 10^−5^
	$\text{Var}(\hat{\text{resid}})$	3.8542× 10^−5^	1.7339× 10^−4^	2.4339× 10^−4^
**Fminor**				
	$\text{Var}(\hat{b_{0}})$	3.1519 × 10^−4^	2.3950 × 10^−1^	3.4108 × 10^−2^
	$\text{Var}(\hat{b_{1}})$	2.9035 × 10^−7^	5.7884 × 10^−5^	1.0166 × 10^−5^
	$\text{Var}(\hat{\text{resid}})$	7.6507× 10^−5^	3.8537× 10^−4^	1.4324× 10^−4^

Visually and numerically, for the given LME model-fitting (Equation 7), this indicates that Spline tracking exhibits a slightly larger “sensitivity” to within-individual variation in time trajectories of mean FA for a tract, compared to DTKODF and DTKTensor tracking.

**Figure 5:**
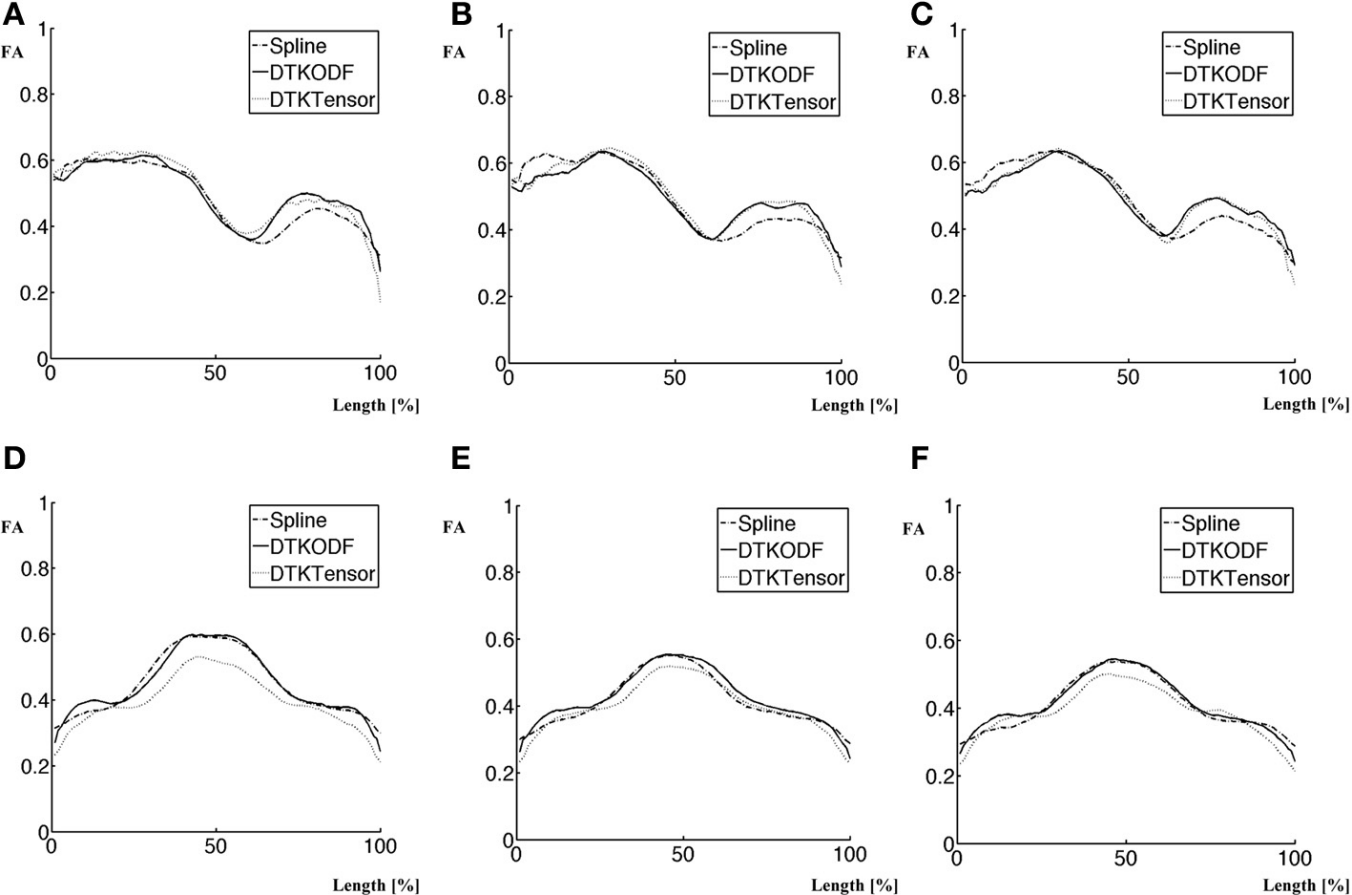
**Along-tract FA values obtained with the three methods: Spline, DTKODF, and DTKTensor. (A)** *CST*—wave1, **(B)** *CST*—wave2, **(C)** *CST*—wave3, **(D)** *Fminor*—wave1, **(E)** *Fminor*—wave2, **(F)** *Fminor*—wave3.

To further explore performance differences between tracking methods, we used along-tract analyses of FA. Comparing the along-tract FA curves in Figure 5, there are at least two interesting observations. Regarding the *CST* (Figures 5A–C), all tracking methods gave two local maxima, at about 30% and about 80%, respectively, of the normalized tract length. Furthermore, our Spline method had the lowest second local maxima.

**Figure 6:**
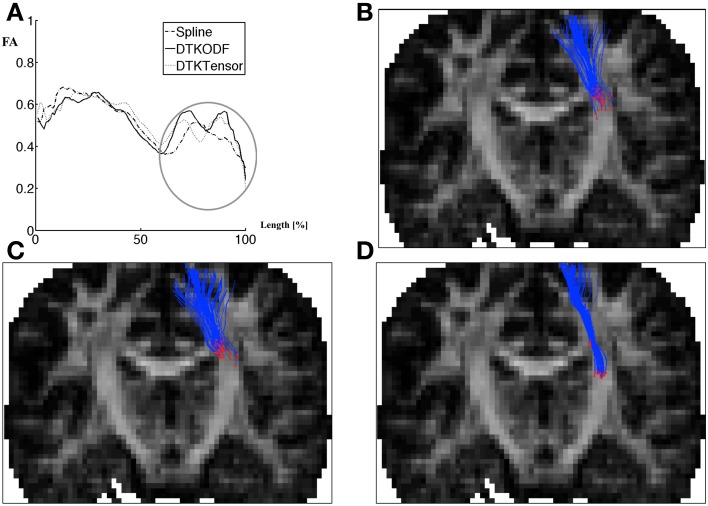
**Part of tract with the largest differences in FA values between the three methods Spline, DTKODF, and DTKTensor, illustrated for one of the subjects. (A)** Along-tract FA curves (wave1) for the left hemisphere *CST*; **(B)** Spline tracking; **(C)** DTKODF tracking; **(D)** DTKTensor tracking. The tracking in **(B–D)** is shown only for the fibers belonging to the final 60–100% segment of the *CST* as indicated by a circle in **(A)**.

**Figure 7:**
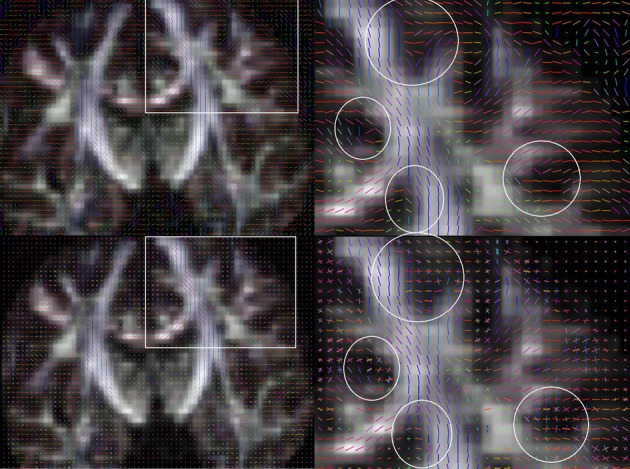
**The differences in the reconstruction techniques applied to one of our datasets, showing the principal eigenvectors of the diffusion tensors (top), and the dODF local maxima (bottom)**. Details of the rectangular inserts are shown to the right. Along the *CST*, there are a few neighboring voxels where the dODF reveals more than one fiber direction. These are indicated by white circles and show that the voxel-wise dODF provides additional information about fiber directions, compared to the diffusion tensor representation.

Figure 6 shows specifically the part of the fiber that is represented in the region of the second local FA maximum (encircled, one subject). Apparently, there is no striking difference between Spline and DTKODF. They both track fibers which are spreading toward the cortex. This is in contrast to the fiber bundle obtained from DTKTensor tracking, which is narrower and is much more spatially restricted. In Figure 7 we have depicted the voxelwise reconstructed fiber directions in a comparable slice, using both tensor (upper panel) and dODF representations (lower panel). For *CST* proper there seems to be only minor differences, but outside the *CST* the dODF helps to reconstruct more (anatomically plausible) fiber directions (circumscribed areas in Figure 7).

Regarding *Fminor*, results from all tracking methods demonstrate very similar curve shape patterns, with one maximum FA between two FA plateaus. The largest difference between the methods is the lower FA values obtained with the DTKTensor tracking.

The tract-averaged fiber analysis was computed by averaging the FA across all fiber coordinates. Additionally, an alternative voxel-averaged fiber analysis was conducted, where each voxel visited by at least one tract was counted only once, and the FA was averaged over these voxels. This approach showed similar results as the tract-averaged fiber analysis.

### Phantom dataset

**Figure 8:**
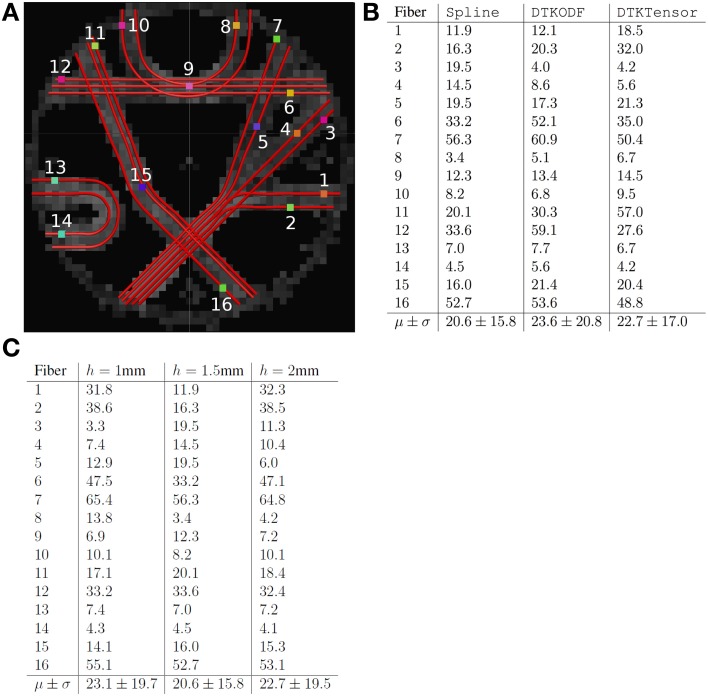
**(A)** Enumerated ground truth fibers in the MICCAI 2009 Fiber Cup phantom dataset. **(B)** Results obtained comparing the different methods Spline, DTKODF, and DTKTensor with the ground truth. **(C)** Results obtained comparing Spline with varying step-length *h* (1, 1.5, and 2 mm). The phantom dataset contains 16 ROIs, and the fibers intersecting each of these ROIs constitute fiber bundle 1–16. For the comparison, the average *spatial metric* using 1000 points were employed (computed with interpolating splines). The lower the metric value, the closer the tracking is to ground truth.

Tracking in the brain phantom dataset was performed in a similar manner as for the human DW-MRI data, except from the step-length *h*, which was set to 1.5 mm. The evaluation of the tracking was done twofold for the phantom. First, we used a dedicated program (fibercup) for comparing the tracking results, available at the web-site of the MICCAI Fiber Cup 2009 (http://www.lnao.fr/spip.php?rubrique79). The coordinates for 16 ground truth fibers are provided by the phantom dataset, in addition to 16 ROIs used to define fibers corresponding to the ground truth. In the fiber tracking method comparison, interpolating splines were used and the fibers were sampled using **s** = 1000 points. A distance measure (**L**^2^) between two corresponding fibers were then computed for these points. The 16 fibers and the ROIs are depicted in Figure 8A. dODFs were computed in TrackVis, and tracking was accomplished by Spline, DTKODF and DTKTensor, respectively. The results from the comparison are presented in Figure 8B. The mean distance (i.e., average value of the **L**^2^ metric estimated over the number of sampling points) between ground truth fibers and the fibers obtained by each of the three tracking methods, is slightly lower for our Spline method (followed by DTKTensor)—in terms of grand mean across the 16 ROIs (last row in Figure 8B). Among the fibers where our Spline method performed better, are the *Fminor*-mimicking tract (ROIs 8, 9, and 10), and also the long-range *CST*-mimicking tract, crossing the midline (ROIs 11, 15, and 16). Inferior results with Spline, however, occurred for tracts defined by ROIs 3 and 4.

The effect of varying the step-length *h* in our Spline method was assessed by performing tracking in the same data set. Figure 8C shows the distance measure (**L**^2^) between the ground truth and our results using *h* = 1 mm, *h* = 1.5 mm, and *h* = 2 mm. The best performance using these three different step-lengths was obtained using *h* = 1.5 mm.

**Figure 9:**
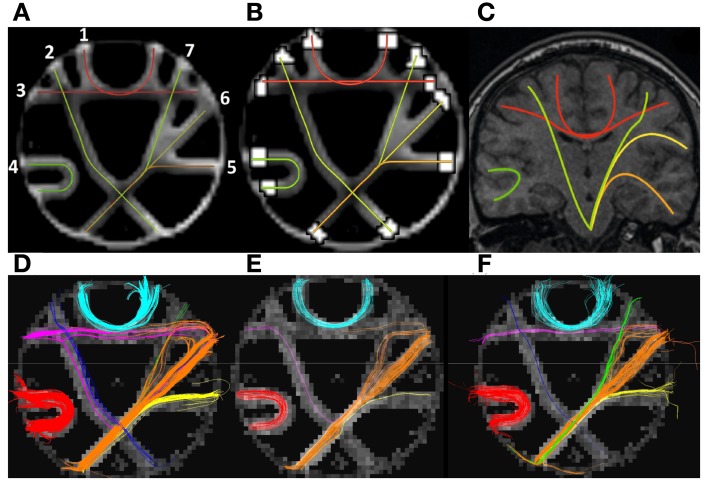
**(A)** The ground truth fibers in the MICCAI 2009 Fiber Cup phantom dataset according to Côte et al. (2012). **(B)** Peripheral ROIs for tract definitions are shown as white areas. **(C)** Coronal brain slice indicating the fibers used as motivation for construction the phantom and its constituent fiber tracts. **(D)** Fibers extracted using our Spline method. **(E)** Fibers extracted using DTKTensor. **(F)** Fibers extracted using DTKODF. **(A–C)** are courtesy of Côte et al. (2012) and tractometer.org.

A different evaluation framework for the phantom data was later proposed by Côte et al. (2012). The ROIs were then re-defined and larger. The number of fibers was reduced to seven, where each fiber was defined by two ROIs (cf. Figures 9A,B), mimicking *in vivo* WM fiber bundles as indicated in Figure 9C. Figures 9D–F, displays the fibers resulting from tracking with Spline **(D)**, DTKTensor **(E)**, and DTKODF **(F)**, connecting the pairs of ROIs shown in Figure 9B, where the underlying dODFs were computed in TrackVis as in the previous evaluation.

**Figure 10:**
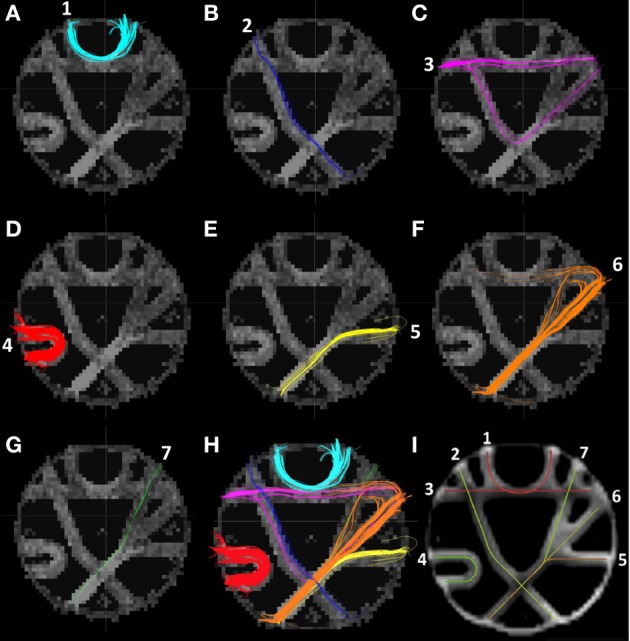
**(A–G)** Individual fibers (1–7) extracted using our Spline method. **(H)** Fibers 1–7 extracted using our Spline method. **(I)** The ground truth fibers in the MICCAI 2009 Fiber Cup phantom dataset according to Côte et al. (2012) [courtesy of Côte et al. (2012) and tractometer.org].

Our Spline tracking method (Figure 9D) was able to find fibers connecting all seven pairs of ROIs, where each of them are visually comparable to the ground truth (Figure 9A). The seven fiber bundles are displayed individually in Figures 10A–G, collectively in Figure 10H, whereas the ground truth fibers are shown in Figure 10I. Two of the fiber bundles deviate somewhat from the ground truth, i.e., bundle 3 (Figure 10C) and 6 (Figure 10F). Our fiber 3 consists of two bundles, one following the correct path of fiber 3 in Figure 10I, the other running down along fiber 2 and up again along fiber 6. A few tracts in fiber 6 bifurcate in the middle segment and continue along fiber 7, before turning laterally, joining the end point ROI of fiber 6. Also, some of the tracts in fiber 6 had a sharp turn close to this ROI, and followed fiber 3 for a short distance. Tracking with DTKODF (Figure 9F) resulted also in fibers connecting 7 out of the 7 pairs of ROIs, all of which are visually comparable to the ground truth fibers. Moreover, this tracking method had similar deficiency as the Spline method in that fiber 6 had a bifurcation with a branch along each of fibers 5 and 7. Finally, the DTKTensor tracking method (Figure 9E) resulted in fiber connections only between 4 out of the 7 pairs of ROIs, as fibers 2, 3, and 7 were partially missing. Thus, for the phantom dataset, the DTKTensor tracking method was clearly inferior to DTKODF and Spline tracking.

## Discussion

The development of HARDI acquisitions and introduction of the orientation distribution function has improved the performance and spatial resolution of *in vivo* investigations of “WM integrity” and the assessment of fiber bundle connections in the brain. However, the new advanced acquisition protocols are time-consuming and most state-of-the-art tracking methods are still solving the Frenet equation using Euler or Runge–Kutta schemes (Basser et al., 2000)—although the tangent tracking vector is frequently taken from the ODFs, rather than the tensor representation when recordings have many diffusion sensitizing directions. Despite these efforts, WM tractography, when applied to clinically feasible DW-MRI acquisitions, is still considered inaccurate with false negative and false positive tracts, and the underlying data seems not fully exploited (Li et al., 2012; Feigl et al., 2013). This opens up for further investigations. We have therefore designed and tested a new tracking method for WM fibers, denoted Spline.

Our tracking method is based on the assumption that gross WM consists of smooth tubular structures (Gössl et al., 2002; Jones, 2011; Catani and de Schotten, 2012). This assumption is partly implicit and partly different from working principles of methods merely solving the Frenet equation. With the traditional approach, one can experience sharp turns arising from noise and inaccuracy in the recorded data and in the derived vector fields. This might lead to termination of the tracking, or to erroneous “jumping” and spurious tracts. Normally, these methods incorporate a smoothing spline applied post-tracking. However, these approximating splines will not influence significantly the path taken by a tract across a region of noisy voxels because the major path has already been decided by the tracking algorithm. Our method was designed to counteract these deficiencies. The tracking is iteratively directed by a smoothing spline, which likely decreases the influence of noisy voxel data on the path of the tract. In our work we have investigated how this strategy affects tracking results in data obtained by clinically applicable recording schemes, and in HARDI data. In both cases, the local water diffusion was chosen to be represented by the previously established dODF as calculated by TrackVis (Wang et al., 2007).

To evaluate the performance of our approach, we compared our results with those obtained with two widely used tracking methods, using both real data and data from a phantom. The phantom data, obtained from the MICCAI 2009 Fiber Cup contest, has a known ground truth regarding its fiber connections and been processed by many competing tracking algorithms (Li et al., 2012; Pontabry et al., 2013). It could therefore be argued that the phantom data alone suffices for the evaluation of our Spline tracking method. Although mimicking a brain coronal slice, the water diffusion properties of the test object may not, however, be representative for water diffusion in white matter tissue of the living human brain. We therefore decided to also include real data in our evaluation, despite the fact that comparative tractography on real data is not straight-forward since the ground truth is not known. Also, post mortem brain and anatomical preparations can be useful in the evaluation of tracking methods (Dyrby et al., 2007; Li et al., 2012; Prats-Galino et al., 2012), but a ground truth is still lacking. On the other side, several WM fiber tracts are typically identified in whole-brain tractography examinations (Bassett et al., 2011; Catani and de Schotten, 2012; Côte et al., 2012; Guevara et al., 2012) and have been subject to extensive studies by many neuroimaging researchers. To better assess the applicability and performance of our Spline tracking method in real life situations, we selected two well-described fiber bundles for the evaluation: the *cortocospinal* tract, and the frontal lobe *forceps minor*. Moreover, these tract evaluations were done on longitudinal data, employing clinical data acquisition schemes. This enabled us to compare tracking algorithms with respect to tract detection/segmentation, and quantification of biological change (in terms of tract-wise FA values) related to aging using linear mixed-effects model fitting.

Large cross-sectional studies involving segmentation of the *CST* have shown a minor reduction in FA values with age (Westlye et al., 2010; Lebel et al., 2012). Westlye et al. (2010) reported a slight FA decline in *CST* from approximately 0.58 at the age of 40 years, to approximately 0.56 for subjects at the age of 80. The study by Lebel et al. (2012) reported a reduction in FA from ~0.52 to ~0.50 for the same age difference. In our experiments with longitudinal data, the tract-averaged FA obtained from eight participants with age range 52–66 years, using Spline, DTKODF and DTKTensor tracking, showed relatively constant *CST* FA during the 6 years of aging (mean FA in the range 0.49–0.50) and therefore supports the conclusions from these studies of near constant mean FA of the *corticospinal tract* into old age. The same investigators (Westlye et al., 2010; Lebel et al., 2012) reported a clear reduction in mean FA of the *Fminor* fiber bundle from 0.55 to 0.47 (Westlye et al., 2010) and from 0.54 to 0.50 (Lebel et al., 2012) between the age span from 40 to 80 years. Our results, using Spline and DTKODF tracking are in accordance with these findings, where we observed a reduction in mean FA of *Fminor* from 0.44 to 0.41 during the 6 years of aging in our sample. The unlikely finding of a slight increase in average FA of *Fminor* from wave1 to wave2 using DTKTensor tracking (Table 1), followed by a slight decrease from wave2 to wave3, although not statistically significant, might be due to noise sensitivity of the local tensor representation and the FACT algorithm implemented in DTKTensor, being highly dependent on a strong principal diffusion direction. Our Spline tracking method and DTKODF tracking, however, demonstrated a pattern of monotonic decrease in mean FA of the *Fminor* tract from wave1 to wave3.

The along-tract analysis revealed some interesting patterns. Firstly, using Spline, DTKODF, and DTKTensor tracking, respectively, the distinct local FA minimum located at approximately 60% along the length-normalized *CST* tract (Figures 5A–C) seems to correspond to the intersection of the *CST* and fibers from the corpus callosum, which could explain the reduced FA due to crossing fibers. This might advocate the use of more reliable and sensitive diffusion parameters, like generalized fractional anisotropy (GFA) (Tuch, 2004; Fritzsche et al., 2010; Jones et al., 2012). We used, however, FA as the diffusion metric since it has been shown to be satisfactory for *b* = 1000 (Fritzsche et al., 2010). All tracking methods resulted in similar FA curves for the first local maximum (at about 30% of trajectory length), but the second local FA maximum (at about 80% trajectory length), was clearly lower for Spline tracking compared to DTKODF and DTKTensor. This local discrepancy between tracking methods was further investigated and Figure 6 shows the 60–100% segment of the length-normalized *CST* for a single subject. By construction, we expect Spline tracking to allow fibers to spread and cover a larger volume than the fibers obtained with DTKTensor tracking. Since FA in general is lower in the outskirts of the *CST*, closer to the cerebral cortex, this would explain the lower local mean FA values along this part of the tract. The local deviation in FA between Spline and DTKODF tracking is harder to explain since the fibers in this segment of *CST* seem to have a similar spreading pattern (Figures 6B,C). However, ground truth is not attainable for the detailed fiber pathways of *CST* (and the corresponding FA values) in a specific brain and region, and no firm conclusion can be made about local accuracy of the tracking methods being compared. Visually, Spline and DTKODF seem to give better results than DTKTensor, since tracking by the latter method produces a rather concentrated and spatially restricted bundle of *CST* fibers toward the cortical sheet which is anatomically less plausible.

Secondly, the along-tract analysis of *Fminor* using Spline, DTKODF and DTKTensor tracking, respectively, gave comparable FA curves with closely corresponding peaks and valleys (Figures 5D–F). However, the DTKTensor tracking produced consistently lower along-tract FA values across the three waves compared to that obtained with the two other methods. To explore whether this was due to difference in tract density or overall volume positioning, we conducted the alternative, voxel-averaged fiber analysis. This confirmed the finding of lower FA along *Fminor* using DTKTensor and indicate that the DTKTensor tracking method applied to *Fminor* will segment a partly different white matter volume than that obtained with the two other tracking methods. Further investigations are thus needed regarding the reduced FA along *Fminor* using DTKTensor tracking.

Along-tract analysis have been used in several recent DWI-MRI studies (Jones et al., 2005; Colby et al., 2012; Hodneland et al., 2012; Yeatman et al., 2012; Hill et al., 2013). In our along-tract analysis of *CST* and *Fminor*, the peaks and valleys of the FA curves were very similar to those reported by Colby et al. (2012, Figure 5). A major difference, however, is that FA in our study was 0.1–0.2 units lower along the *CST* than that reported in Colby et al. (2012). For *Fminor*, the along-tract FA discrepancy was much less (<0.1 units in lower values), where the Spline and the DTKODF tracking methods produced FA curves closest to those in Colby and coworkers. It should be noted that the along-tract curves reported in Colby et al. (2012, Figure 5) are from a single subject, which can explain the discrepancies in FA. Furthermore, the DWI-MRI acquisitions in Colby et al. (2012) were performed on a 3T scanner, with different pulse sequences, and with voxel-wise tensor representations, also giving possible rise to the observed bias in FA.

For evaluation of tracking performance on the brain phantom data, two different strategies were adopted. Applying the first strategy (Fillard et al., 2011), we showed an improved fiber tracking ability of Spline, compared to the DTKODF and DTKTensor methods. Comparing our results with those from the MICCAI 2009 Fiber Cup contest, our Spline tracking method could not entirely match the best results. However, it should be noted that some of the methods being used in this contest included a fiber bundle selection (e.g., Reisert et al., 2009) where only a selected subset of the resulting fibers were used in the evaluation. Following the second strategy (Côte et al., 2012), we achieved good results with Spline and DTKODF. Both methods were able to find 7 out of 7 fibers, whereas DTKTensor was able to reconstruct only 5 out of 7 fibers. We suspect the poorer results with DTKTensor to be due to its voxel-wise diffusion tensor representation, instead of dODFs as in DTKODF and Spline, misleading the tracking in regions with crossing fibers. This observation supports the use of dODFs rather than DTs for practical tracking applications.

There have been only few reports (e.g., Prckovska et al., 2010) on the use of ODF to DWI-MRI recordings with a low to moderate angular resolution, i.e., 20–30 diffusion sensitizing directions. We have in this work demonstrated that the orientation distribution function can be better suited to represent regions of crossing fibers than using a local tensor representation—both for the brain phantom data and for (clinical) data with relatively few (<30) diffusion sensitizing directions. In Figure 7 we have depicted the voxelwise reconstructed fiber directions in a slice from one of the subjects in the longitudinal study, using both tensor (upper panel) and dODF representations (lower panel). Most studies with DWI-MRI data similar to ours have been using diffusion tensor fiber tracking (cf. DTKtensor). This study indicate that dODF reconstruction can be employed even at low angular resolution of 25 diffusion sensitizing directions recorded within clinically feasible acquisition times.

## Summary and conclusion

We have proposed a new tracking method (Spline) as an alternative to the traditional streamline tracking method based on the Frenet equations. This is done by imposing a contextual constraint via an iterative smoothing spline, using voxel-wise estimation of the diffusion orientation distribution function, rather than a tensor representation. The motivation and design principle of Spline was to improve the resistance against erroneous tracking in noisy voxels. The other major contribution is the incorporation of a comprehensive evaluation framework for comparing tracking methods, spanning both real DWI-MRI recordings (longitudinal data with linear mixed-effects model fitting), detailed along-tract analysis of diffusion parameters (FA), and the use of DWI-MRI acquisitions from a test object, mimicking a brain, with ground truth for its fiber connections. Using this evaluation framework, we compared Spline with two other tracking methods (DTKODF and DTKtensor) provided in widely used software for DWI-MRI analysis. We found some major differences between Spline and two other tracking methods, that might have practical implications. This regards results from along-tract analyses of fractional anisotropy, in particular for the *corticospinal tract* and also for the *forceps minor*. Testing our method on data from the phantom with a ground truth, the performance of our method was generally better than the two widely used tracking methods being compared.

We find these design considerations and results encouraging, and further algorithmic improvements and evaluation procedures are planned. Additional metrics and evaluation strategies are explained in Fillard et al. (2011), Côte et al. (2012), and the use of these would provide a more thorough evaluation of our results. To our disposal we also have DW-MRI data from nearly 100 participants across three study waves obtained in the aforementioned study of aging, genetics and cognition (Westlye et al., 2011; Ystad et al., 2011; Hodneland et al., 2012). Moreover, it would be important to study and reconstruct a wider collection of fiber tracts, other than the *CST* and *Fminor*, in order to quantify regional differences that can be attributed to the aging process, and to better clarify the influence of tracking method being used. For such longitudinal investigations, where also results from cognitive testing will be included, proper statistical methods are essential, and linear and non-linear mixed-effects models should be methods of choice.

### Conflict of interest statement

The authors declare that the research was conducted in the absence of any commercial or financial relationships that could be construed as a potential conflict of interest.

## References

[B1] Basser P. LeBihan D. (1992). Fiber orientation mapping in an anisotropic medium with NMR diffusion spectroscopy, in Society for Magnetic Resonance in Medicine (SMRM) Proceedings, 1992. Vol. 11 (Berlin), 1121

[B2] Basser P. Mattiello J. LeBihan D. (1992). Diagonal and off-diagonal components of the self-diffusion tensor: their relation to and estimation from the NMR spin-echo signal, in Society for Magnetic Resonance in Medicine (SMRM) Proceedings, 1992. Vol. 11 (Berlin), 1221

[B3] Basser P. J. Pajevic S. Pierpaoli C. Duda J. Aldroubi A. (2000). *in vivo* fiber tractography using DT-MRI data. Magn. Reson. Med. 44, 625–632 10.1002/1522-2594(200010)44:4<625::AID-MRM17>3.0.CO;2-O 11025519

[B4] Bassett D. S. Brown J. A. Deshpande V. Carlson J. M. Grafton S. T. (2011). Conserved and variable architecture of human white matter connectivity. Neuroimage 54, 1262–1279 10.1016/j.neuroimage.2010.09.006 20850551

[B5] Besseling R. M. H. Jansen J. F. A. Overvliet G. M. Vaessen M. J. Braakman H. M. H. Hofman P. A. M. (2012). Tract specific reproducibility of tractography based morphology and diffusion metrics. PLoS ONE 7:e34125 10.1371/journal.pone.0034125 22485157 PMC3317780

[B6] Campbell J. S. W. Siddiqi K. Rymar V. V. Sadikot A. F. Pike G. B. (2005). Flow-based fiber tracking with diffusion tensor and Q-ball data: validation and comparison to principal diffusion direction techniques. Neuroimage 27, 725–736 10.1016/j.neuroimage.2005.05.014 16111897

[B7] Catani M. de Schotten M. T. (2012). Atlas of Human Brain Connections. Oxford: Oxford University Press 10.1093/med/9780199541164.001.0001

[B8] Colby J. B. Soderberg L. Lebel C. Dinov I. D. Thompson P. M. Sowell E. R. (2012). Along-tract statistics allow for enhanced tractography analysis. Neuroimage 59, 3227–3242 10.1016/j.neuroimage.2011.11.004 22094644 PMC3288584

[B9] Côte M. Boré A. Girard G. Houde J. C. Descoteaux M. (2012). Tractometer: online evaluation system for tractography, in MICCAI 2012, Part I. LNCS 7510 (Berlin: Springer-Verlag), 699–706 10.1007/978-3-642-33415-3_86 23285613

[B10] Descoteaux M. Angelino E. Fitzgibbons S. Deriche R. (2007). Regularized, fast, and robust analytical Q-ball imaging. Magn. Reson. Med. 58, 497–510 10.1002/mrm.21277 17763358

[B11] Descoteaux M. Deriche R. Knösche T. R. Anwander A. (2009). Deterministic and probabilistic tractography based on complex fibre orientation distributions. IEEE Trans. Med. Imaging 28, 269–286 10.1109/TMI.2008.2004424 19188114

[B12] Dyrby T. B. Søgaard L. V. Parker G. J. Alexander D. C. Lind N. M. Baare W. F. C. (2007). Validation of *in vitro* probabilistic tractography. Neuroimage 37, 1267–1277 10.1016/j.neuroimage.2007.06.022 17706434

[B13] Feigl G. C. Hiergeist W. Fellner C. Schebesch K.-M. M. Doenitz C. Finkenzeller T. (2013). Magnetic resonance imaging diffusion tensor tractography: evaluation of anatomic accuracy of different fiber tracking software packages. World Neurosurg. [Epub ahead of print]. 10.1016/j.wneu.2013.01.004 23295636

[B14] Fernandez-Miranda J. C. Pathak S. Engh J. Jarbo K. Verstynen T. Yeh F. (2012). High-definition fiber tractography of the human brain: neuroanatomical validation and neurosurgical applications. Neurosurgery 71, 430–453 10.1227/NEU.0b013e3182592faa 22513841

[B15] Fillard P. Descoteaux M. Goh A. Gouttard S. Jeurissen B. Malcolm J. (2011). Quantitative evaluation of 10 tractography algorithms on a realistic diffusion MR phantom. Neuroimage 56, 220–234 10.1016/j.neuroimage.2011.01.032 21256221

[B16] Fritzsche K. H. Laun F. B. Meinzer H.-P. Stieltjes B. (2010). Opportunities and pitfalls in the quantification of fiber integrity: what can we gain from Q-ball imaging? Neuroimage 51, 242–251 10.1016/j.neuroimage.2010.02.007 20149879

[B17] Gerald C. F. Wheatley P. O. (1999). Applied Numerical Analysis. Reading, MA: Addison Wesley Longman

[B18] Gössl C. Fahrmeir L. Pütz B. Auer L. M. Auer D. P. (2002). Fiber tracking from DTI using linear state space models: detectability of the pyramidal tract. Neuroimage 16, 378–388 10.1006/nimg.2002.1055 12030823

[B19] Guevara P. Duclap D. Poupon C. Marrakchi-Kacem L. Fillard P. Le Bihan D. (2012). Automatic fiber bundle segmentation in massive tractography datasets using a multi-subject bundle atlas. Neuroimage 61, 1083–1099 10.1016/j.neuroimage.2012.02.071 22414992

[B20] Hill S. Y. Terwilliger R. McDermott M. (2013). White matter microstructure, alcohol exposure, and familial risk for alcohol dependence. Psychiatry Res. 212, 43–53 10.1016/j.pscychresns.2012.11.003 23473988 PMC3714312

[B21] Hodneland E. Ystad M. Haász J. Munthe-Kaas A. Lundervold A. (2012). Automated approaches for analysis of multimodal MRI acquisitions in a study of cognitive aging. Comput. Methods Programs Biomed. 106, 328–341 10.1016/j.cmpb.2011.03.010 21663993

[B22] Jenkinson M. Smith S. (2001). A global optimisation method for robust affine registration of brain images. Med. Image. Anal. 5, 143–156 10.1016/S1361-8415(01)00036-6 11516708

[B23] Jeurissen B. Leemans A. Tournier J.-D. Jones D. K. Sijbers J. (2012). Investigating the prevalence of complex fiber configurations in white matter tissue with diffusion magnetic resonance imaging. Hum. Brain Mapp. [Epub ahead of print]. 10.1002/hbm.22099 22611035 PMC6870534

[B24] Jones D. K. (ed.). (2011). Diffusion MRI. New York, NY: Oxford University Press

[B25] Jones D. K. Knösche T. R. Turner R. (2012). White matter integrity, fiber count, and other fallacies: the do's and don'ts of diffusion MRI. Neuroimage 73, 239–254 10.1016/j.neuroimage.2012.06.081 22846632

[B26] Jones D. K. Travis A. R. Eden G. Pierpaoli C. Basser P. J. (2005). PASTA: pointwise assessment of streamline tractography attributes. Magn. Reson. Med. 53, 1462–1467 10.1002/mrm.20484 15906294

[B27] Lebel C. Gee M. Camicioli R. Wieler M. Martin W. Beaulieu C. (2012). Diffusion tensor imaging of white matter tract evolution over the lifespan. Neuroimage 60, 340–352 10.1016/j.neuroimage.2011.11.094 22178809

[B28] Li L. Rilling J. K. Preuss T. M. Glasser M. F. Damen F. W. Hu X. (2012). Quantitative assessment of a framework for creating anatomical brain networks via global tractography. Neuroimage 61, 1017–1030 10.1016/j.neuroimage.2012.03.071 22484406 PMC3407566

[B29] Losnegård A. Lundervold A. Haasz J. Hodneland E. (2011). Fast marching tractography from multiple diffusion sensitizing directions in MR-DTI from the brain,” in Proceedings of the 7th International Symposium on Image and Signal Processing and Analysis (ISPA) 2011 (Dubrovnik), 497–500

[B30] Madden D. J. Bennett I. J. Burzynska A. Potter G. G. Chen N.-K. Song A. W. (2012). Diffusion tensor imaging of cerebral white matter integrity in cognitive aging. Biochim. Biophys. Acta 1822, 386–400 10.1016/j.bbadis.2011.08.003 21871957 PMC3241892

[B31] Mori S. Crain B. Zijl P. (1998). 3D brain fiber reconstruction from diffusion MRI, in Proceedings of International Conference on Functional Mapping of the Human Brain, 1998 (Montreal).

[B32] Mori S. van Zijl P. C. M. (2002). Fiber tracking: principles and strategies – a technical review. NMR Biomed. 15, 468–480 10.1002/nbm.781 12489096

[B33] Parker G. J. M. Wheeler-Kingshott C. A. M. Barker G. J. (2002). Estimating distributed anatomical connectivity using fast marching methods and diffusion tensor imaging. IEEE Trans. Med. Imaging 21, 505–512 10.1109/TMI.2002.1009386 12071621

[B34] Pontabry J. Rousseau F. Oubel E. Studholme C. Koob M. Dietemann J.-L. (2013). Probabilistic tractography using q-ball imaging and particle filtering: application to adult and *in-utero* fetal brain studies. Med. Image. Anal. 17, 297–310 10.1016/j.media.2012.11.004 23265801

[B35] Poupon C. Laribere L. Tournier G. Bernard J. Fournier D. Fillard P. (2010). A diffusion hardware phantom looking like a coronal brain slice, in Proceedings of the International Society for Magnetic Resonance in Medicine, 2010 (Stockholm).

[B36] Poupon C. Rieul B. Kezele I. Perrin M. Poupon F. Mangin J.-F. (2008). New diffusion phantoms dedicated to the study and validation of high-angular-resolution diffusion imaging (HARDI) models. Magn. Reson. Med. 60, 1276–1283 10.1002/mrm.21789 19030160

[B37] Prats-Galino A. Soria G. de Notaris M. Puig J. Pedraza S. (2012). Functional anatomy of subcortical circuits issuing from or integrating at the human brainstem. Clin. Neurophysiol. 123, 4–12 10.1016/j.clinph.2011.06.035 22055838

[B38] Prckovska V. Rodrigues P. Duits R. Haar Romeny B. T. Vilanova A. (2010). Extrapolating fiber crossings from DTI data. Can we gain similar information as HARDI?” in Workshop on Computational Diffusion MRI, MICCAI, 2010 (Beijing), 26–37

[B39] Reisert M. Mader I. Anastasopoulos C. Weigel M. Schnell S. Kiselev V. (2011). Global fiber reconstruction becomes practical. Neuroimage 54, 955–962 10.1016/j.neuroimage.2010.09.016 20854913

[B40] Reisert M. Mader I. Kiselev V. (2009). Tracking a physical phantom by global fibre reconstruction, in MICCAI 2009, Fiber Cup 2009 (London, UK).

[B41] Stejskal E. O. Tanner J. E. (1965). Spin diffusion measurements: spin echoes in the presence of a time-dependent field gradient. J. Chem. Phys. 42, 924–937 10.1063/1.1695690

[B42] Tuch D. Wiegell M. Reese T. Belliveau J. Wedeen V. (2001). Measuring cortico-cortical connectivity matrices with diffusion spectrum imaging, in Proceedings in International Society of Magnetic Resonance in Medicine (ISMRM), 2001. Vol. 502 (Glasgow, UK).

[B43] Tuch D. S. (2004). Q-ball imaging. Magn. Reson. Med. 52, 1358–1372 10.1002/mrm.20279 15562495

[B44] Wakana S. Caprihan A. Panzenboeck M. M. Fallon J. H. Perry M. Gollub R. L. (2007). Reproducibility of quantitative tractography methods applied to cerebral white matter. Neuroimage 36, 630–644 10.1002/mrm.20279 17481925 PMC2350213

[B45] Wang R. Benner T. Sorensen A. G. Wedeen V. (2007). Diffusion toolkit: a software package for diffusion imaging data processing and tractography. Proc. Intl. Soc. Mag. Reson. Med. 15, 3720

[B46] Wedeen V. J. Reese T. G. Tuch D. S. Wiegell M. R. Dou J. G. Weiskoff R. M. (2000). Mapping fiber orientation spectra in cerebral white matter with Fourier-transform diffusion MRI. Proc. Intl. Sot. Mag. Reson. Med. 8, 82 16247738

[B47] Wedeen V. J. Wang R. P. Schmahmann J. D. Benner T. Tseng W. Y. I. Dai G. (2008). Diffusion spectrum magnetic resonance imaging (DSI) tractography of crossing fibers. Neuroimage 41, 1267–1277 10.1016/j.neuroimage.2008.03.036 18495497

[B48] Westin C.-F. Maier S. E. Khidhir P. P. E. Jolesz F. Kikinis R. (1999). Image processing for diffusion tensor magnetic resonance imaging, in Medical Image Computing and Computer Assisted Intervention (MICCAI) Proceedings, 1999 (Cambridge, UK), 441–452

[B49] Westlye E. T. Lundervold A. Rootwelt H. Lundervold A. J. Westlye L. T. (2011). Increased hippocampal default mode synchronization during rest in middle-aged and elderly APOE ε4 carriers: relationships with memory performance. J. Neurosci. 31, 7775–7783 10.1523/JNEUROSCI.1230-11.2011 21613490 PMC6633142

[B50] Westlye L. T. Walhovd K. B. Dale A. M. Bjørnerud A. Due-Tønnessen P. Engvig A. (2010). Life-span changes of the human brain white matter: diffusion tensor imaging (DTI) and volumetry. Cereb. Cortex 20, 2055–2068 10.1093/cercor/bhp280 20032062

[B51] Yeatman J. D. Dougherty R. F. Myall N. J. Wandell B. A. Feldman H. M. (2012). Tract profiles of white matter properties: automating fiber-tract quantification. PLoS ONE 7:e49790 10.1371/journal.pone.0049790 23166771 PMC3498174

[B52] Yeh F.-C. Wedeen V. J. Tseng W.-Y. I. (2010). Generalized q-sampling imaging. IEEE Trans. Med. Imaging 29, 1626–1635 10.1109/TMI.2010.2045126 20304721

[B53] Ystad M. Hodneland E. Adolfsdottir S. Haász J. Lundervold A. J. Eichele T. (2011). Cortico-striatal connectivity and cognition in normal aging: a combined DTI and resting state fMRI study. Neuroimage 55, 24–31 10.1016/j.neuroimage.2010.11.016 21073962

